# Methylation of Host Genes Associated with Coronavirus Infection from Birth to 26 Years

**DOI:** 10.3390/genes12081198

**Published:** 2021-07-31

**Authors:** Rutu Rathod, Aniruddha Rathod, Parnian Kheirkhah Rahimabad, Jiasong Duan, Hongmei Zhang, S. Hasan Arshad, Wilfried Karmaus

**Affiliations:** 1 Division of Epidemiology, Biostatistics, and Environmental Health Sciences, School of Public Health, University of Memphis, Memphis, TN 38111, USA; rpatel15@memphis.edu (R.R.); abrathod@memphis.edu (A.R.); Parnian.K@memphis.edu (P.K.R.); jduan@memphis.edu (J.D.); hzhang6@memphis.edu (H.Z.); 2 Clinical and Experimental Sciences, Faculty of Medicine, University of Southampton, Southampton SO17 1BJ, UK; S.H.Arshad@soton.ac.uk; 3 David Hide Asthma and Allergy Research Centre, Isle of Wight, Newport PO30 5TG, UK; 4 NIHR Southampton Biomedical Research Centre, University Hospital Southampton, Southampton SO17 1BJ, UK

**Keywords:** coronavirus, DNA methylation, SARS-CoV2, COVID-19, epigenetics, IoW cohort

## Abstract

DNA methylation (DNAm) patterns over time at 1146 CpGs on coronavirus-related genes were assessed to understand whether the varying differences in susceptibility, symptoms, and the outcomes of the SARS-CoV-2 infection in children and young adults could be explained through epigenetic alterations in a host cell’s transcriptional apparatus to coronaviruses. DNAm data from the Isle of Wight birth cohort (IOWBC) at birth, 10, 18, and 26 years of age were included. Linear mixed models with repeated measurements stratified by sex were used to examine temporal patterns, and cluster analysis was performed to identify CpGs following similar patterns. CpGs on autosomes and sex chromosomes were analyzed separately. The association of identified CpGs and expression of their genes were evaluated. Pathway enrichment analyses of the genes was conducted at FDR = 0.05. DNAm at 635 of the 1146 CpGs on autosomes showed statistically significant time effects (FDR = 0.05). The 635 CpGs were classified into five clusters with each representing a unique temporal pattern of DNAm. Of the 29 CpGs on sex chromosomes, DNAm at seven CpGs in males and eight CpGs in females showed time effects (FDR = 0.05). Sex-specific and non-specific associations of DNAm with gene expression were found at 24 and 93 CpGs, respectively. Genes which mapped the 643 CpGs represent 460 biological processes. We suggest that the observed variability in DNAm with advancing age may partially explain differing susceptibility, disease severity, and mortality of coronavirus infections among different age groups.

## 1. Introduction

Coronaviruses are a group of zoonotic pathogens that were previously considered to cause relatively benign infections in humans [1]. However, the emergence of severe acute respiratory syndrome coronavirus (SARS-CoV) in 2002 in China, and the Middle East respiratory syndrome coronavirus (MERS-CoV) in 2012 in Middle Eastern countries, proved coronaviruses’ ability to cause severe respiratory disorders [1]. Recently, for the third time, a novel coronavirus called SARS-CoV-2 crossed the interspecies barrier and caused a global health crisis [2].

Epidemiological studies have shown a significant difference between adult and pediatric populations in terms of incidence and symptomatology of SARS-CoV-2 infection. Children seem to be less susceptible to develop the disease and are more likely to present with milder symptoms if infected, while adult subjects are prone to develop severe forms with higher mortality rates [3]. Data from around the world suggests that COVID-19 susceptibility [4], positivity [4,5,6,7,8], hospitalization [6], and mortality rates [9] increases with age, i.e., individuals at younger age are at lower risk compared to those at older age. Studies on SARS and MERS infections also show a similar trend of milder symptoms and lower mortality rates in children compared to adults [10,11]. Recent studies have identified the role of host factors at a molecular level to explain the gap. Children and adults have shown different distribution and functioning of angiotensin-converting enzyme-2 (ACE2), the receptor coded on the X-chromosome and used by the SARS virus, SARS-CoV-2, and human coronavirus-NL63 (HCoV-NL63) [12]. In addition, cellular and molecular components, of both the innate and adaptive immune systems, and their ability to coordinate an effective immune response deteriorates drastically with age, explaining poorer outcomes in the elderly [13].

Although an individual’s genetic information is stable, its epigenetics can change significantly over time. Growing evidence revealed epigenetics, particularly DNA methylation (DNAm) at cytosine-phosphate-guanine (CpG) sites, to be one of the crucial mechanisms underlying the aging process [14]. The importance of epigenetics and DNAm have been lately emphasized in the pathogenesis of several viral infections, including coronaviruses [15,16,17,18]. DNAm is an important regulator that alters host expression patterns. These changes have important implications for the activity of the virus itself since it relies on the host cell to replicate its genetic material and continue to proliferate [18,19,20]. Recently, Corley et al. reported differential DNAm at CpGs associated with *ACE2* in distinct age groups, possibly explaining the gap of SARS-CoV2 infection risk in children and adults [21]. Given the dynamic nature of DNAm during aging and its potential role in coronavirus infection, we suggest that changes in DNAm over time could explain the differences in susceptibility, symptoms, and the outcomes of the SARS-CoV-2 infection in children and young adults. In this study, we assessed DNAm levels of CpGs on candidate genes that were assigned to be associated with SARS CoV-2 infections from birth to 26 years using the Isle of Wight (IoW) birth cohort. To differentiate between systematic age-related and random variation of DNAm, we compared the candidate gene DNAm over the years with those in housekeeping and other immune-related genes. We also assessed the association of identified dynamic DNAm with expression of their mapped genes at 26 years. The results of our study may contribute to a better understanding of the underlying mechanisms of differences in hosts’ susceptibility and responses to SARS-CoV-2 over time. In addition, epigenetic targets may be identified for the future preventive and therapeutic measures.

## 2. Materials and Methods

### 2.1. Study Population

The Isle of Wight birth cohort (IoWBC) was established to study natural history of allergic disorders in a semi-rural island near the UK mainland [22]. Children born from 1 January 1989 to 28 February 1990 were included in the cohort. From the 1536 pregnancies in this period, 1456 parents with live births consented for recruitment and follow-up at 1, 2, 4, 10, 18 and 26 years. Demographic information was obtained using hospital records at birth and detailed questionnaires provided at each follow-up session. Blood samples were collected for DNAm measurement at birth (Guthrie cards) and at 10, 18, and 26 years (peripheral blood).

### 2.2. Coronavirus-Related Genes and CpGs

Genes potentially related to SARS-CoV-2 pathogenesis were identified from GeneCards (www.genecards.org/ accessed on 28 March 2020) [23] using the keywords ‘Coronavirus’ and ‘Coronavirus silent sweep’, i.e., genes associated with silent infection of coronavirus leading to improved immunity resulting in a subclinical disease. The genes were selected for the study based on the score for each gene obtained from GeneCards, i.e., the number of times that a gene was shown to be associated with coronavirus in the literature. In particular, a scree plot of the scores in descending order was implemented and genes with scores showing large decreases before flattening out were selected. CpGs on these genes were identified using the Illumina manifestation file and were included in the analysis of this study. Additionally, information on the chromosomes that these CpGs are on, their locations relative to CpG islands, and locations on genes were extracted from the manifestation file.

### 2.3. Housekeeping and Immune Genes and Their CpGs

Five housekeeping (HK) genes were randomly identified that are not known to be related to immunity, coronavirus, or silent sweep infection. Additionally, genes potentially related to immunity were identified from GeneCards using the keyword ‘immune’, and five immune genes were randomly selected that are not known to be related to coronavirus or silent sweep infection. These HK and immune-related genes were selected to compare their DNAm patterns with patterns seen in coronavirus-related genes.

### 2.4. DNA Methylation (DNAm)

DNA was extracted from Guthrie cards (blood collected within 5 days of birth) using a procedure previously described by Beyan et al. [24], and from peripheral blood samples at 10, 18 and 26 years using a standard salting out procedure [25]. DNA concentration was determined by PicoGreen dsDNA quantitation (Molecular Probes, INC. Eugene, OR, USA) or Qubit (Thermofisher, Waltham, MA, USA). For each sample, about 1 µg DNA was treated with bisulfite to convert cytosine to thymine using the EZ 96-DNAm Kit (Zymo Research, Irvine, CA, USA). DNAm levels were assessed using the Infinium HumanMethylation450 BeadChips and MethylationEPIC BeadChips (Illumina, Inc., San Diego, CA, USA) using a standard protocol [26] arrays that were processed with multiple identical control samples allocated to each bisulfite-converted batch to determine assay variability. A BeadStation was used to scan the beadchips. DNAm level, i.e., the β value for each queried CpG locus, was assessed using BeadStudio software (Methylation module).

CPACOR pipeline was used for quality control of the DNAm data [27]. Specifically, the DNA methylation intensity data were quantile-normalized using the R package, *minfi* [28], and ComBat was applied to remove the batch effects [29]. DNAm levels are presented in β values calculated using proportions of intensity of methylated (*M*) over the sum of methylated and unmethylated (*U*) sites (*β* = *M* ∕ [c +*M* + *U*], where c is a constant to prevent division by zero if *M* + *U* is too small). Since β values ranged between 0 and 1, a base-2 logit transformation was applied to β values (denoted as M values) to avoid severe heteroscedasticity [30]. CpGs with probe-SNPs within ten base pairs and with minor allele frequency (MAF) greater than 0.007 were excluded as they may influence DNA methylation measurements. CpGs on the sex chromosomes were analyzed separately from CpGs on autosomes and were stratified by sex.

Since blood is composed of functionally and developmentally different cell populations [31], we adjusted the cell type proportions to remove the potential confounding effect of cell heterogeneity in DNAm measured from blood samples [32]. Cell type proportions were estimated using the Bioconductor *minfi* package [22], a method proposed by Jaffe and Irizarry [27] and adapted from Houseman et al. [28]. We included the estimated cell type proportions as adjusting factors in the analyses.

### 2.5. Genome-Wide RNA-Seq Gene Expression Data Generation

Peripheral blood samples obtained at 26 years were used for the assessment of gene expression levels using paired-end (2 × 75 bp) RNA sequencing with the Illumina Tru-Seq Stranded mRNA Library Preparation Kit with IDT for Illumina Unique Dual Index (UDI) barcode primers according to the manufacturer’s instructions. We sequenced all samples twice using the same protocol and combined the output from both runs. The quality of the FASTQ files (https://www.bioinformatics.babraham.ac.uk/projects/fastqc/ accessed on 8 May 2021) were assessed by running FASTQC. We mapped the reads against human genome (GRch37 version 75) using HISAT2 (v2.1.0) aligner [33]. The sequence alignment map (SAM) format produced the alignment files which were subsequently converted into the Binary Alignment Map (BAM) format using SAMtools (v1.3.1) [34]. The reads mapped to each gene were counted using HTseq (v0.11.1) in the same reference genome used for alignment [35]. We used the countToFPKM package (https://github.com/AAlhendi1707/countToFPKM accessed on 8 May 2021) to calculate the normalized read count fragments per kilobase of transcript per million mapped reads (FPKM) which were further log-transformed and used in the data analysis.

### 2.6. Statistical Analysis

Linear mixed models with repeated measurements were implemented to characterize trends in CpG sites, such that DNAm levels changed over time from birth to adulthood. DNAm of CpG sites expressed as M-values at birth, 10, 18, and 26 years were included in the model as the dependent variable and time (with birth as the reference group) and gender (with males as the reference group) as predictors. The estimated cell-type proportions of CD4+ T cells, natural killer cells, neutrophils, B cells, monocytes, and eosinophils were included in the analyses as adjusting factors. CpGs with statistically significant time effects were considered as being dynamic CpGs. Multiple testing was corrected on the overall F tests on time effects by controlling for FDR at 0.05 level. An identical analysis was performed for CpGs of HK and immune-related genes.

Cluster analysis was performed on the regression coefficients at 10, 18, and 26 years to identify CpGs showing similar patterns (trajectories) in DNAm over time using proc fastclus (SAS). Three to ten clusters were assessed, and the highest pseudo-F statistic and cubic clustering criterion were selected to decide on the number of clusters. The profile of each cluster was visualized using cluster medians of regression coefficients at birth, 10, 18, and 26 years to display the group patterns. CpGs on sex chromosome were analyzed separately using the same statistical analysis, stratified by sex. The analysis was performed in SAS version 9.4.

The biologic significance of the identified dynamic CpGs were assessed by evaluating the associations between gene expression of their mapped genes and DNAm at 26 years. Linear regressions were used where gene expression (*n* = 139) was the outcome, and DNAm (in M-values) and sex were the exposure variables. An interaction term of DNAm × sex was included in the model as we previously have found the association between gene expression and DNAm to be different in both males and females [36]. Statistical significance of the interaction effects was set at *p*-value < 0.05. If the interaction was not statistically significant, the main effects of DNAm were evaluated after adjusting for sex. For CpGs on sex chromosomes, whose values may vary by the number of X chromosomes, the analysis was stratified by sex.

### 2.7. Pathway Enrichment Analyses

Genes annotated to the dynamic CpGs were identified based on the Illumina manifest file. Pathway enrichment analysis of the genes was conducted using Toppfun (https://toppgene.cchmc.org/enrichment.jsp accessed on 24 July 2021). Additionally, we performed two more pathway analysis using candidate genes and non-dynamic candidate genes (i.e., genes with CpGs not showing dynamic patterns) to compare biological processes with dynamic genes with whole genome as a background. Multiple testing was adjusted by controlling Bonferroni *p*-value of 0.05.

## 3. Results

From GeneCards, genes with a score of >2.8 (the top 66 genes) were selected based on a scree plot for coronavirus and 14 genes for coronavirus silent sweep infection. In total, 1555 CpGs located on these 78 genes were extracted from the Illumina manifestation file. Of these, four CpGs with ‘ch’ prefix were excluded, and DNAm of the remaining 1551 CpGs at different time points (i.e., birth, 10, 18, 26 years) were analyzed. Of the 1551 CpGs, 29 CpGs were found to be located on sex chromosome on gene *ACE2* and *CD40LG*, and the analysis was stratified by gender for those CpGs.

Of 1522 CpGs on autosomes, DNAm data was available for 988 CpGs at birth (*n* = 796), 688 CpGs at 10 years (*n* = 330), 688 CpGs at 18 years (*n* = 476), and 677 CpGs at 26 years (*n* = 242), in the IoW cohort (in total, 1146 CpGs). Identified through use of linear mixed models, DNAm at 635 of the 1146 CpGs (55.4%) showed statistically significant time effects at the FDR = 0.05 level (Table 1 and Supplement Table S1a). We observed both linear and non-linear time effects on DNAm. The pattern of these CpGs/genes was called dynamic. For 100 identified CpGs on five randomly selected HK genes, DNAm data was available for 47 CpGs at birth, 40 CpGs at 10 years, 40 CpGs at 18 years, and 33 CpGs at 26 years (58 CpGs in total). DNAm at only one of the 58 CpGs showed significant time effects at the FDR = 0.05 level (1.72%, Supplement Table S1b). For 125 identified CpGs on five randomly selected immune genes, DNAm data were available for 69 CpGs at birth, 41 CpGs at 10 years, 41 CpGs at 18 years, and 39 CpGs at 26 years (76 CpGs in total). DNAm at 11 of the 76 CpGs showed significant time effects at the FDR = 0.05 level (14.47%, Supplement Table S1c, Supplement Figure S1).

**Figure 1:**
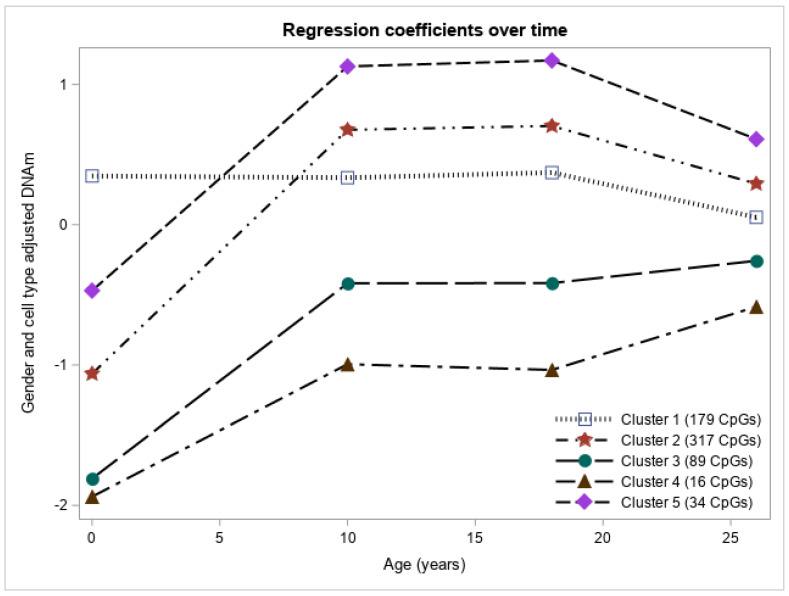
DNA methylation profiles of the five CpG clusters represented by the median of gender and cell type heterogeneity adjusted DNA methylation at each age.

For each CpG site, results from linear mixed models enabled us to estimate DNAm at each time point with gender and cell type heterogeneity adjusted. These adjusted DNAm were then used to cluster the CpG sites to reveal different temporal DNAm patterns. Based on the pseudo-F statistic and cubic clustering criterion, we grouped the 635 CpG sites into five clusters (Figure 1). In clusters 2 (317 CpGs) and 5 (34 CpGs), DNAm on average showed similar patterns over time (increase from birth to 10 years, minimal changes from 10 to 18 years, and slight decrease from 18 to 26 years), but DNAm at CpGs in cluster 5 tends to be higher than that in cluster 2. Average DNAm patterns in clusters 3 (89 CpGs) and 4 (16 CpGs) also showed similar patterns over time (increase from birth to 10 years, minimal changes from 10 to 18 years, and an increase from 18 to 26 years) with cluster 3 having a higher DNAm on average. The temporal pattern of average DNAm in the CpG cluster 1 is unique in that it is stable overall with a slight decrease from 18 to 26 years. In terms of average DNAm across all the five clusters, DNAm levels were lower on average for CpGs in clusters 3 and 4 compared to CpGs in clusters 2 and 5, while average DNAm in cluster 1 was in the middle starting from age 10 but the highest at birth (Figure 1).

**Figure 2:**
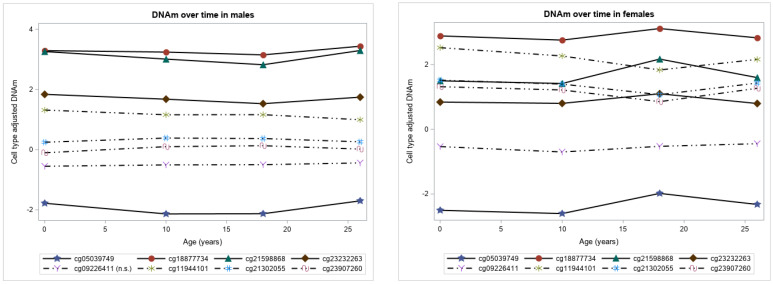
DNA methylation patterns of the available CpGs on sex chromosomes in males and females represented by cell type adjusted DNA methylation over time. The solid lines represent temporal patterns of CpGs on gene *ACE2* and dash-dotted lines are for CpGs on gene *CD40LG*.

Of the 29 CpGs on sex chromosome, DNAm data were available for eight CpGs at birth, 10, 18, and 26 years (*n* = 506 males, 506 females). In linear mixed models, statistically significant time effects were observed at seven CpGs in males and eight CpGs in females after adjusting for multiple testing at the FDR = 0.05 level (Table 2, Figure 2).

The association between the identified dynamic CpGs and expression of their mapped genes were assessed. Significant effects for the interaction of DNAm and sex were observed at 24 CpGs with 15 genes on autosomes (all *p*-values <4.48 × 10^−2^. Table 3 and Supplement Table S2a). Of the 24 CpGs, the estimates for both sexes were in opposite direction at 22 CpGs (i.e., opposite signs of regression coefficients after combing the main and interaction effects), thereby suggesting a potential gender reversal at these specific CpG sites. For example, with one unit increase in DNAm levels of cg21657705, the expression of *ACE* gene is downregulated by 0.64 units in males while it upregulates *ACE* expression by 0.45 units (=1.09–0.64) in females (Table 3 and Supplement Table S2a). At 16 of the 22 CpGs, an increase in DNAm was associated with decreased expression in females while there were increased gene expression levels in males. Whereas, at 6 of the 22 CpGs, an increase in DNAm was associated with increased expression in females but decreased gene expression in males. For CpGs without interaction effects, main effects were assessed and significant association of 93 CpGs with 31 genes were found after adjusting for sex (all *p*-values <5 × 10^−2^. Table 4 and Supplement Table S2b). The models evaluating the association of DNAm and gene expression on sex chromosomes were stratified by sex. An increase in DNAm at CpG site cg23907260 was associated with increased gene expression levels of *CD40LG* in males (estimate: 0.55, *p*-value: 8.40 × 10^−3^. Supplement Table S2c).

The 643 identified dynamic CpGs (showing statistically significant time effects) were mapped to 60 genes (referred to as dynamic genes). To better understand the biological function of these 643 CpGs, pathway enrichment analyses was conducted for the 60 dynamic genes, 78 candidate genes, and 18 (=78–60) non-dynamic candidate genes. Using these genes in *ToppFun*, with the whole genome as the background, we identified 460, 524, and 38 biological processes for dynamic, candidate, and non-dynamic candidate genes, respectively, which were enriched after multiple testing adjusted by controlling for Bonferroni *p*-value of 0.05 (Table 5 and Supplement Table S3).

## 4. Discussion

We examined the development of methylation levels of CpGs potentially associated with coronavirus infection using four measurements spanning from birth to age 26 years. DNAm levels at 635 CpGs on autosomes showed significant time effects. For most of the CpGs on autosomes, increasing age was associated with a rise in DNAm levels from birth to pre-adolescence period, no change in DNAm levels from pre- to post-adolescence, and a decrease in DNAm levels from post-adolescence to adulthood. About 55.4% (=635/1146) of CpGs on coronavirus-related genes were identified as dynamic compared to 1.72% (=1/58) in random samples of HK genes and 14.47% (=11/76) of immune-related genes. In addition, the temporal patterns in DNAm were consistent across all the identified dynamic CpGs on immune-related genes, which were different from the patterns revealed by the dynamic CpGs on coronavirus-related genes. These findings suggest that the observed variability in DNAm levels with advancing age may, in part, explain differing susceptibility, disease severity, and mortality of coronavirus infections among distinct age groups. More specifically, lower DNAm levels at most of CpGs from birth to pre-adolescence compared to other ages may provide protection against SARS-CoV-2 infection. An increase in DNAm levels at pre-adolescence and no change from pre- to post-adolescence might explain the increase in the number of coronavirus cases from 10 to 18 years compared to earlier ages. From post-adolescence to adulthood, DNAm levels decrease at some CpGs and increase at some CpGs, which may explain high susceptibility, morbidity, and mortality among adults compared to children. The sex chromosome, DNAm, at seven CpGs (four on *ACE2* and three on *CD40LG*) in males and eight CpGs (four on *ACE2* and four on *CD40LG*) in females showed significant time effects.

In this study, we observed DNAm levels of CpGs on the *ACE2* gene to be higher in males compared to females, while on *CD40LG* gene, DNAm levels were higher in females compared to males from birth to adulthood. It has been shown previously that *CD40LG* (CD40 ligand) on the X chromosome is involved in response to infections and escapes X inactivation in some cells, contributing to gender differences in immune responses [37]. In addition, the identified CpGs on autosomes and sex chromosomes suggest that there is a possibility of epigenetic regulation on gene activities, and that the observed sex-specific associations of gene expression and DNAm at some CpGs may be linked to the observed gender gap in incidence and mortality of coronaviruses, although in-depth assessment is certainly needed.

The CCL5 gene on autosome drew most of our attention. One of the CpG site on this gene showed the most statistically significant change over time (with *p*-value at the level of 10^−308^). In addition, DNAm at multiple CpGs on this gene was shown to be associated with expression of CCL5. In particular, three CpGs (cg10315334, cg02867514, cg08656816) have the strongest biological relevance among all the CpGs identified, reflected by their largest effects on gene expression as well as the smallest *p*-values, regardless of sex. The CCL5 gene has been previously linked to the susceptibility and pathogenesis of SARS-CoV [38,39] and SARS-CoV-2 [40,41].

Recently, Corley et al. showed DNAm levels of CpGs associated with ACE2 in airway epithelial cells to be age-dependent, potentially explaining the host differences in children and adults [21]. Our findings indicated that age-dependency of DNAm at CpGs on this gene was also present in blood. However, since ACE2 is not expressed in blood [42,43,44,45], we were not able to assess the biological relevance of the identified CpGs.

The pathways identified using dynamic candidate and non-dynamic candidate genes in the pathway analysis yielded a different number of biological processes. Of the 460 biological processes identified based on dynamic genes, 429 were unique compared to the processes identified based on non-dynamic genes, indicating different underlying biological functionalities between these two sets of genes. Pathway enrichment analysis revealed biological processes involved in host immune function (e.g., immune effector process, cellular response to cytokine stimulus, cytokine-mediated signaling pathway, response to biotic stimulus, cytokine, other organism, external biotic stimulus,) and the response to viruses (e.g., viral process, defense response to virus and other organism). These biological processes have been suggested to have critical roles in the pathogenesis of coronavirus infections [46,47,48]. Lymph nodes maintain and coordinate new immune responses to control the viruses, although age-related lymph nodes changes reduce the ability of B and T cells to proliferate and differentiate in lymph nodes. Because of this reason, new immune responses are blunted, with significantly less effector cells that are less well prepared by antimicrobial molecules. It is assumed that this mechanism in older adults renders them less effective in defending against SARS-CoV-2 infection [13]. Diao. et al. demonstrated a reduction and functional exhaustion of surviving T cells in COVID-19 patients [49]. Lymphocytes are significantly reduced in the SARS-CoV-2 infected subjects which is directly affected by the viral load [50], possibly due to SARS-CoV-2-induced activation of apoptosis [51]. It was shown that there is an association of COVID-19 pathogenesis and excessive cytokine release [51]. Issa et al. identified six functional domains (I to VI) in the SARS-CoV-2 3a protein that were linked to virulence, infectivity, ion channel formation, and virus release in SARS-CoV-2 [52]. In addition, immune response differences in children compared to adults includes lower production of proinflammatory cytokines, higher production of immunomodulatory cytokines, decreased infiltration of neutrophils, and a predominance of CD4+ T cells [53]. These pathophysiological differences in children and adults are believed to underlie lower susceptibility of children to coronavirus infections and their diminished immune mediated lung injury [53].

The strength of this study is the availability of DNAm from birth to 26 years, enabling us to examine changes from birth to adulthood for CpGs on autosomes and sex chromosome separately. To our knowledge, this is the first study to examine the epigenetics of genes potentially associated with the coronavirus to explain differences in susceptibility, morbidity, and mortality among children and adults. Our effort was to try to explain the susceptibility, morbidity, and mortality through DNAm levels in children and adults. However, we were unable to directly assess changes in DNAm among subjects infected with coronavirus due to the lack of COVID-19 data. It would also be preferable to look at the individual DNAm patterns (trajectories), including older adults; however, to our knowledge, no cohort currently has DNAm data covering birth, childhood, and early and late adulthood. In our study, DNA was extracted from peripheral blood cells. Coronaviruses affect many different cell types, primarily cells of the respiratory tract [54]. In our analysis, we extracted candidate genes from the literature, potentially related to SARS-CoV-2 infection, and did not have information on tissues or cells which these genes were identified in. It has been shown that DNAm of blood cells has concordance with that of the respiratory system cells [55], although some differences exist between the two. Almost all coronavirus genes included in the study are immune-related genes; however, not all immune-related genes are coronavirus-related genes. Future studies linking gene with alterations of methylation are needed to directly assess association of DNAm with coronavirus infections in older adults and elderly populations. Large cohorts with DNAm data before and after the recent coronavirus pandemic can be used to (a) estimate risks related to differences in DNAm of the exposed subjects who did and did not develop the infection, (b) estimate differences in DNAm of the patients who presented with mild, moderate, or severe symptoms, and (c) demonstrate the effects of the infection on the epigenome of the host. Our findings showed that changes in DNAm levels from birth to adulthood in some genes might explain COVID-19 susceptibility and severity differences in children and young adults. Thus, our results are informative for the COVID-19 disease map [56], as it provides details on possible involvement in changing susceptibility to the coronavirus based on DNAm of various genes.

## Figures and Tables

**Table 1:** DNAm for CpGs on autosomes showing significant time effects (dynamic) along with the information on locations of the identified CpG sites. Only results for top 10 CpGs showing the most statistically significant time effects at FDR = 0.05 level was shown.

CpG Site	F Value	FDR *p*-Value	Gene	Chromosome Number	CpG Islands	Gene Location
cg04791421	1218.77	4.15 × 10^−308^	*CCL5*	17		TSS1500
cg21278129	1176.06	9.26 × 10^−303^	*CLEC4G*	19	S_Shore	TSS1500
cg11694510	1127.39	1.85 × 10^−296^	*IFITM1*	11	S_Shore	TSS1500
cg03589230	1031.87	2.2 × 10^−283^	*MYOM2*	8		TSS1500
cg21686213	1000.14	7.48 × 10^−279^	*IFITM1*	11	N_Shore	3′UTR
cg20545776	972.67	7.67 × 10^−275^	*GPT*	8	N_Shore	TSS1500
cg12876900	957.15	1.51 × 10^−272^	*IFITM3*	11	S_Shore	1st Exon
cg16628205	922.08	3.25 × 10^−267^	*TFR2*	7		TSS1500
cg14231966	914.28	4.97 × 10^−266^	*FURIN*	15	Island	Body
cg03038262	907.51	5.37 × 10^−265^	*IFITM1*	11	N_Shore	3′UTR

**Table 2:** DNAm for CpGs on sex chromosomes showing significant time effects (dynamic) along with the information on locations of the identified CpG sites. The analysis was stratified by sex.

CpG Site	F Value	FDR *p*-Value	Gene	Gene Location	Sex
cg21598868	63.77	4.64 × 10^−35^	*ACE2*	TSS1500	Females
cg05039749	44.64	1.31 × 10^−25^	*ACE2*	Body	Females
cg23232263	38.77	1.33 × 10^−22^	*ACE2*	3′UTR	Females
cg18877734	22.13	1.78 × 10^−13^	*ACE2*	TSS1500	Females
cg11944101	97.5	1.22 × 10^−49^	*CD40LG*		Females
cg23907260	91.36	2.56 × 10^−47^	*CD40LG*	TSS200	Females
cg21302055	77.44	2.53 × 10^−41^	*CD40LG*	TSS200	Females
cg09226411	18.16	3.01 × 10^−11^	*CD40LG*	TSS200	Females
cg21598868	22.59	3.78 × 10^−13^	*ACE2*	TSS1500	Males
cg05039749	26.73	3.58 × 10^−15^	*ACE2*	Body	Males
cg23232263	13.81	2.26 × 10^−8^	*ACE2*	3′UTR	Males
cg18877734	7.99	3.72 × 10^−5^	*ACE2*	TSS1500	Males
cg11944101	19.18	2.23 × 10^−11^	*CD40LG*		Males
cg23907260	11.63	3.53 × 10^−7^	*CD40LG*	TSS200	Males
cg21302055	8.14	3.52 × 10^−5^	*CD40LG*	TSS200	Males
cg09226411	1.9	0.13	*CD40LG*	TSS200	Males

**Table 3:** Sex-specific associations of DNAm with expression of their mapped genes. Five CpGs with the most statistically significant interaction effects of DNAm × sex on gene expression were shown. Males are in the reference group. The *p*-values are for interaction effects. The full list of CpGs showing significant interaction effects (24 CpGs mapped to 15 genes) are in Supplement Table S2a.

CpG	Gene	DNAm Effect	Sex × DNAm Interaction Effect	*p*-Value (Sex × DNAm)	Gene Location
cg12455187	*CCL5*	1.25	−1.38	7.00 × 10^−4^	TSS1500
cg20559158	*MYOM2*	1.81	−2.94	8.42 × 10^−3^	Body
cg00162643	*DDX58*	−0.68	0.83	7.07 × 10^−3^	TSS200
cg15096505	*IL10*	−3.17	2.30	3.11 × 10^−3^	Body
cg21657705	*ACE*	−0.64	1.09	2.82 × 10^−3^	Body

**Table 4:** Sex-unspecific associations of DNAm with expression of their mapped genes. Five CpGs with the most statistically significant main effects (sex unspecific) of DNAm on gene expression are shown. Males are in the reference group. The full list of CpGs showing significant interaction effects (93 CpGs mapped to 31 genes) are in Supplement Table S2b.

CpG	Gene	DNAm Effect	*p*-Value	Gene Location
cg10315334	*CCL5*	−1.79	2.98 × 10^−17^	1st Exon, 5′UTR
cg02867514	*CCL5*	−1.62	6.88 × 10^−15^	1st Exon, 5′UTR
cg08656816	*CCL5*	−1.72	6.03 × 10^−15^	TSS200
cg15055101	*SH2D3A*	−1.31	3.69 × 10^−14^	5′UTR
cg15353603	*DPP4*	−0.90	5.01 × 10^−10^	Body

**Table 5:** Top 10 most significant biological processes from pathway enrichment analysis of dynamic and non-dynamic candidate genes.

Dynamic Genes		Non-Dynamic Candidate Genes	
Biological Processes	Bonferroni *p*-Value	Biological Processes	Bonferroni *p*-Value
**immune effector process**	1.17 × 10^−24^	response to cytokine	1.85 × 10^−5^
defense response	1.34 × 10^−24^	defense response	5.74 × 10^−5^
response to biotic stimulus	9.40 × 10^−24^	cell activation	1.66 × 10^−4^
response to cytokine	1.45 × 10^−20^	cellular response to cytokine stimulus	1.81 × 10^−4^
response to other organism	2.41 × 10^−20^	entry into host	2.51 × 10^−4^
response to external biotic stimulus	2.51 × 10^−20^	response to other organism	2.57 × 10^−4^
cellular response to cytokine stimulus	4.59 × 10^−19^	response to external biotic stimulus	2.60 × 10^−4^
cytokine-mediated signaling pathway	1.63 × 10^−18^	response to biotic stimulus	3.22 × 10^−4^
**defense response to other organism**	3.82 × 10^−18^	movement in host environment	4.83 × 10^−4^
viral process	9.63 × 10^−17^	viral life cycle	4.85 × 10^−4^

Note: Two biological processes in bold font are unique to dynamic genes, i.e., were not obtained in pathways of non-dynamic candidate genes.

## Data Availability

The data presented in this study are available on request from the corresponding author. The data are not publicly available because the cohort was established with specific focus on asthma and allergy related studies. The data access is restricted to ensure that the use of data is in compliance with participants’ consent and ethics approval.

## Supplementary Materials

The following are available online at https://www.mdpi.com/article/10.3390/genes12081198/s1, Table S1a: DNAm for CpGs on autosomes of coronavirus-related genes showing significant time effects along with the information on locations of the identified CpG sites, Table S1b: DNAm for CpGs on housekeeping genes showing significant time effects along with the information on locations of the identified CpG sites, Table S1c: DNAm for CpGs on immune-related genes showing significant time effects along with the information on locations of the identified CpG sites, Table S2a: Association of DNAm at 24 CpGs with their mapped genes’ expression levels on autosomes that are sex-specific. Only results on CpGs showing statistically significant interaction effects of DNAm × sex on gene expression were shown. Males are in the reference group. The *p*-values are for interaction effects, Table S2b: Association of DNAm at 93 CpGs with their mapped genes’ expression levels on autosomes that are sex-nonspecific. Only results on CpGs showing statistically significant DNAm effects on gene expression were shown. Males are in the reference group, Table S2c: Association of DNAm with their mapped genes’ expression levels on sex chromosomes, Table S3: Significant GO terms and its biological processes from pathway enrichment analysis of genes annotated to the dynamic CpGs, Figure S1: DNA methylation (DNAm) levels of the 11 CpGs on immune-related genes at each age demonstrates consistent temporal patterns at all the 11 CpGs (i.e., parallel to each other) except for cg17498272 which remains roughly the same over time.
