# Supplementary material for: Methylation of Host Genes Associated with Coronavirus Infection from Birth to 26 Years
Source: Genes (Basel). 2021 Jul 31;12(8):1198. doi: 10.3390/genes12081198 (PMC8392033; doi:10.3390/genes12081198)
Supplement: Supplementary file 1 [file genes-12-01198-s001.zip › Supplement figure S1.pdf]

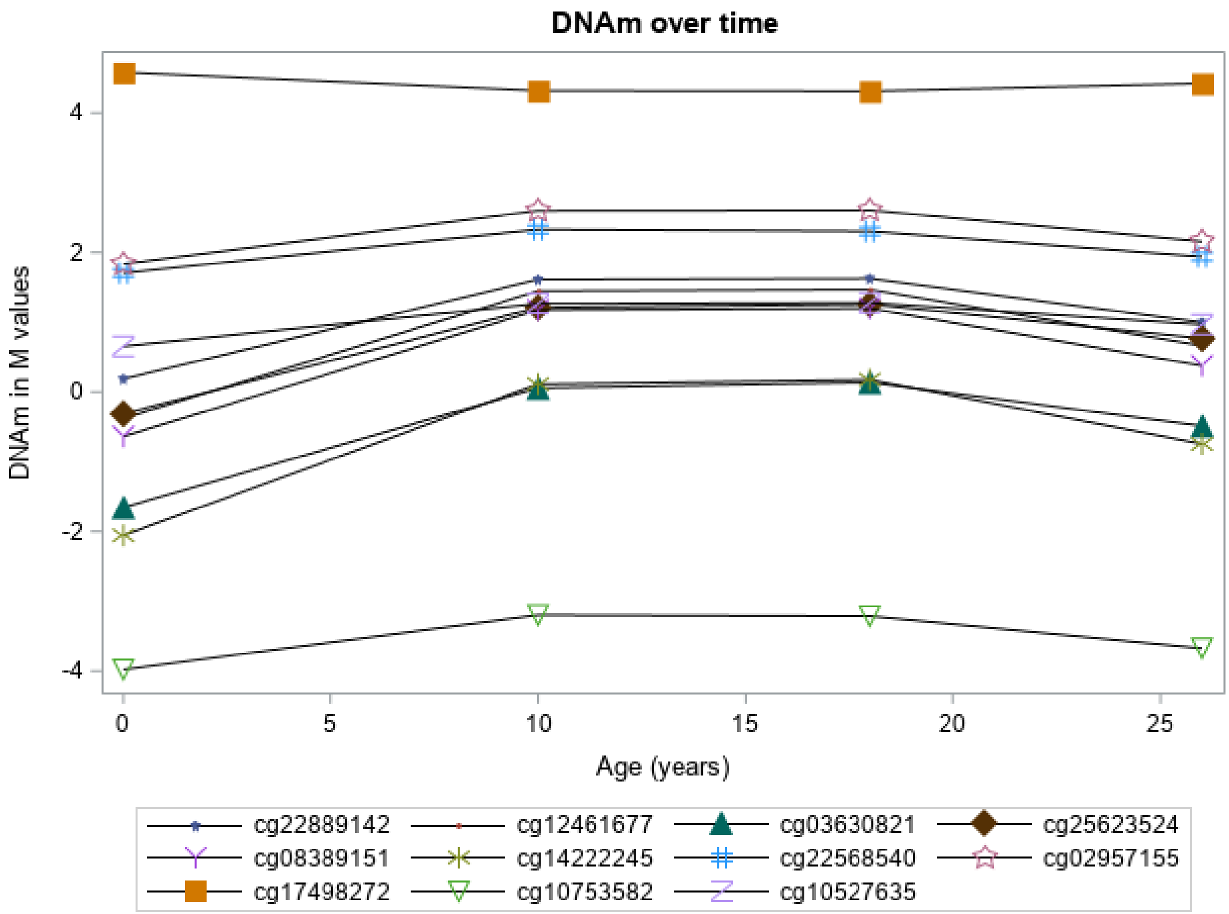

Supplement figure S1. *DNA methylation (DNAm) levels of the 11 CpGs on immune-related genes at each age demonstrates consistent temporal patterns at all the 11 CpGs (i.e., parallel to each other) except for cg17498272 which remains roughly the same over time.*
