# Supplementary material for: Methylation of Host Genes Associated with Coronavirus Infection from Birth to 26 Years
Source: Genes (Basel). 2021 Jul 31;12(8):1198. doi: 10.3390/genes12081198 (PMC8392033; doi:10.3390/genes12081198)
Supplement: Supplementary file 1 [file genes-12-01198-s001.zip › Supplement table S2.pdf]

Table S2a. Association of DNAm at 24 CpGs with their mapped genes' expression levels on autosomes that are sex-specific. Only results on CpGs showing statistically significant interaction effects of DNAm×sex on gene expression were shown. Males are in the reference group. The p-values are for interaction effects.

CpG	Gene	DNAm effect	Sex×DNAm interaction effect	p-value (Sex×DNAm)	Sample size
cg21657705	*ACE*	-0.64	1.09	2.82×10^{-3}	139
cg12455187	*CCL5*	1.25	-1.38	7.00×10^{-4}	139
cg19411729	*CCL5*	-2.07	0.59	4.25×10^{-2}	139
cg00162643	*DDX58*	-0.68	0.83	7.07×10^{-3}	139
cg24354612	*ICAM1*	0.11	-0.53	4.12×10^{-2}	139
cg14145194	*ICAM3*	-0.25	0.71	2.12×10^{-2}	139
cg02935305	*MAPK14*	0.11	-0.44	4.20×10^{-2}	139
cg26974281	*MAPK14*	0.78	-0.92	1.11×10^{-2}	139
cg09726469	*MCL1*	0.80	-1.07	1.37×10^{-2}	139
cg00840694	*MYOM2*	1.03	-2.04	3.19×10^{-2}	139
cg15456293	*MYOM2*	2.02	-2.27	3.27×10^{-2}	139
cg20219329	*MYOM2*	0.80	-1.60	3.09×10^{-2}	139
cg20559158	*MYOM2*	1.81	-2.94	8.42×10^{-3}	139
cg20638896	*MYOM2*	-2.11	2.60	3.25×10^{-2}	139
cg21844893	*MYOM2*	2.95	-3.74	1.28×10^{-2}	139
cg25541958	*MYOM2*	1.54	-3.58	3.27×10^{-2}	139
cg11373557	*POLD1*	-0.45	0.63	1.70×10^{-2}	139
cg09369818	*TFR2*	0.36	-0.40	2.24×10^{-2}	139
cg11860203	*CCL2*	0.72	-2.74	4.48×10^{-2}	130
cg27038101	*CTRL*	2.12	-2.50	3.20×10^{-2}	67
cg04038163	*CXCL9*	0.45	-1.09	4.03×10^{-2}	133
cg15096505	*IL10*	-3.17	2.30	3.11×10^{-3}	132
cg17744604	*IL10*	-1.38	1.41	1.86×10^{-2}	132
cg02335517	*IL6*	0.58	-1.62	2.01×10^{-2}	106

For instance, with one unit increase in DNAm levels of cg21657705, the expression of *ACE* gene is downregulated by 0.64 units in males while it upregulates *ACE* expression by 0.45 units (=1.09-0.64) in females.

Table S2b. Association of DNAm at 93 CpGs with their mapped genes' expression levels on autosomes that are sex-nonspecific. Only results on CpGs showing statistically significant DNAm effects on gene expression were shown. Males are in the reference group.

CpG	Gene	DNAm effect	P-value	Sample size
cg02261408	*ACE*	0.68	3.05×10^{-2}	139
cg10468385	*ACE*	-0.43	1.54×10^{-2}	139
cg19802564	*ACE*	-0.65	1.13×10^{-2}	139
cg20622019	*ADA*	-0.35	2.22×10^{-4}	139
cg05312779	*ANPEP*	0.67	1.10×10^{-3}	139
cg05985767	*ANPEP*	0.44	1.58×10^{-3}	139
cg06963233	*ANPEP*	-0.35	4.52×10^{-3}	139
cg07096901	*ANPEP*	0.45	4.30×10^{-3}	139
cg12382398	*ANPEP*	-0.42	4.51×10^{-4}	139
cg19405555	*ANPEP*	-0.24	4.79×10^{-2}	139
cg23161492	*ANPEP*	-0.38	1.83×10^{-3}	139
cg07679836	*BAK1*	-0.55	1.52×10^{-9}	139
cg24505687	*BAK1*	-0.57	3.11×10^{-8}	139
cg00447324	*CCL5*	-0.88	1.93×10^{-6}	139
cg02483931	*CCL5*	-0.75	2.90×10^{-3}	139
cg02867514	*CCL5*	-1.62	6.88×10^{-15}	139
cg08656816	*CCL5*	-1.72	6.03×10^{-15}	139
cg10315334	*CCL5*	-1.79	2.98×10^{-17}	139
cg11811510	*CEACAM1*	-0.50	2.14×10^{-4}	139
cg19776453	*CEACAM1*	-1.12	6.56×10^{-4}	139
cg05626117	*CLEC4G*	0.64	1.06×10^{-3}	139
cg05842480	*CLEC4G*	0.81	2.83×10^{-2}	139
cg21693780	*DDX1*	-0.30	4.02×10^{-2}	139
cg14286514	*DDX58*	0.29	1.20×10^{-2}	139
cg12649175	*DPP4*	0.50	2.42×10^{-2}	139
cg15353603	*DPP4*	-0.90	5.01×10^{-10}	139
cg20053454	*DPP4*	-0.47	6.67×10^{-3}	139
cg20427486	*DPP4*	-0.79	6.38×10^{-8}	139
cg04946721	*ERN1*	-0.29	1.85×10^{-2}	139
cg20998539	*ERN1*	-0.90	5.22×10^{-3}	139
cg12015851	*FURIN*	0.17	1.49×10^{-2}	139
cg12075901	*FURIN*	-0.23	1.28×10^{-2}	139
cg19348484	*FURIN*	-0.27	4.43×10^{-4}	139
cg06334471	*HELLS*	-0.26	1.25×10^{-2}	139
cg07489134	*HELLS*	-0.59	1.18×10^{-2}	139
cg02776148	*ICAM1*	0.26	1.38×10^{-2}	139
cg13981319	*ICAM3*	-0.27	1.96×10^{-2}	139

cg26972937	*ICAM3*	0.59	1.05×10^{-2}	139
cg06046490	*IFITM3*	-0.72	2.42×10^{-6}	139
cg08596817	*IFITM3*	-0.98	6.33×10^{-7}	139
cg20008734	*IFITM3*	-1.11	3.98×10^{-7}	139
cg21052403	*IFITM3*	-1.44	5.46×10^{-5}	139
cg24897127	*IFITM3*	-0.64	1.56×10^{-2}	139
cg18517766	*IRF3*	0.33	5.52×10^{-3}	139
cg05495029	*BCL2L12*	-0.42	1.93×10^{-3}	139
cg21035241	*BCL2L12*	-0.47	9.89×10^{-4}	139
cg05495029	*IRF3*	-0.37	1.84×10^{-3}	139
cg06490385	*IRF3*	-0.25	6.46×10^{-3}	139
cg21035241	*IRF3*	-0.37	2.67×10^{-3}	139
cg16145324	*MAPK14*	-0.27	9.98×10^{-3}	139
cg17498313	*MAPK14*	0.38	1.52×10^{-2}	139
cg17327331	*MPP5*	0.79	1.49×10^{-4}	139
cg13507964	*MX1*	-0.74	6.91×10^{-3}	139
cg16785077	*MX1*	-1.25	6.72×10^{-4}	139
cg21549285	*MX1*	-1.05	1.58×10^{-7}	139
cg22862003	*MX1*	-1.63	4.40×10^{-7}	139
cg26312951	*MX1*	-1.45	9.67×10^{-7}	139
cg00319223	*MYOM2*	0.78	2.88×10^{-2}	139
cg00527552	*MYOM2*	1.35	6.48×10^{-3}	139
cg00535538	*MYOM2*	-2.24	4.88×10^{-7}	139
cg06445972	*MYOM2*	1.35	4.16×10^{-2}	139
cg14696926	*MYOM2*	-0.64	4.37×10^{-2}	139
cg19307543	*MYOM2*	-1.18	2.86×10^{-6}	139
cg21816685	*MYOM2*	0.71	3.35×10^{-2}	139
cg00420742	*NLRP12*	-0.30	2.89×10^{-2}	139
cg22337438	*NLRP12*	-0.39	1.05×10^{-3}	139
cg00450979	*POLD1*	0.22	1.55×10^{-2}	139
cg11964938	*POLD1*	-0.30	2.69×10^{-2}	139
cg08300117	*PPIA*	0.28	2.18×10^{-2}	139
cg26307266	*PPIA*	-0.21	3.63×10^{-2}	139
cg01036779	*SH2D3A*	-0.57	2.26×10^{-3}	139
cg09962952	*SH2D3A*	0.26	1.73×10^{-2}	139
cg14530382	*SH2D3A*	0.37	2.09×10^{-9}	139
cg15055101	*SH2D3A*	-1.31	3.69×10^{-14}	139
cg01919768	*TFR2*	-0.35	1.15×10^{-2}	139
cg03808577	*TFR2*	0.47	2.37×10^{-2}	139
cg04002480	*TFR2*	-0.47	6.95×10^{-4}	139
cg04423314	*TFR2*	0.48	2.87×10^{-4}	139
cg10681065	*TFR2*	0.63	4.37×10^{-5}	139
cg01360627	*TNF*	-0.43	9.83×10^{-7}	139

cg04472685	*TNF*	-0.41	2.26×10^{-3}	139
cg15989608	*TNF*	-0.59	8.24×10^{-7}	139
cg20477259	*TNF*	-0.54	4.57×10^{-6}	139
cg23384708	*TNF*	-0.56	1.19×10^{-5}	139
cg24452282	*TNF*	-0.48	1.35×10^{-5}	139
cg26736341	*TNF*	-0.59	3.58×10^{-6}	139
cg13495204	*ALB*	-0.63	5.00×10^{-2}	93
cg02185216	*GPT*	0.48	1.69×10^{-2}	105
cg20545776	*GPT*	1.86	7.36×10^{-4}	105
cg00848007	*IFNG*	-0.76	8.92×10^{-4}	126
cg01281450	*IFNG*	-1.20	2.23×10^{-4}	126
cg01940810	*IFNG*	-1.22	4.30×10^{-8}	126
cg26227465	*IFNG*	-1.44	1.04×10^{-7}	126

Table S2c. Association of DNAm with their mapped genes' expression levels on sex chromosomes

**Gender**	**CpG**	**Gene**	**DNAm effect**	**P-value**	**Sample size**
Male	cg23907260	*CD40LG*	0.55	8.40×10^{-3}	139
